# Low-dose oral flecainide provocation test for Brugada syndrome: a case series

**DOI:** 10.1186/s43044-025-00679-3

**Published:** 2025-10-07

**Authors:** Xuan Nguyen Thanh, Tuan Ngoc Tran, Thuan Nguyen Van, Nguyen Nguyen Duc, Thao Pham Ngoc, Hanh Nguyen Thi, Hoi Nguyen Van, Luyen Nguyen Van

**Affiliations:** 1 Military Hospital 103, Vietnam Military Medical University, Hanoi, Viet Nam; 2 Vietnam Military Medical University, Hanoi, Viet Nam; 3 Vietnam Military Medical University, Hanoi, Viet Nam

**Keywords:** Brugada syndrome, Flecainide challenge, Provocation test

## Abstract

**Background:**

Brugada syndrome is a rare, inherited cardiac disorder that predisposes individuals to life-threatening ventricular arrhythmias, often leading to sudden cardiac arrest. In many cases, the characteristic electrocardiographic (ECG) findings of Brugada syndrome are not present at baseline but can be unmasked using sodium channel blockers. While intravenous ajmaline is the preferred agent, its limited availability has led to the increased use of oral flecainide for provocation testing. Previous studies have used 300–400 mg doses, but the efficacy and safety of a lower dose, such as 200 mg, have not been systematically evaluated. This report presents three cases demonstrating that a 200 mg oral flecainide dose may be sufficient to unmask the diagnostic Type 1 Brugada ECG pattern in selected patients.

**Case presentation:**

Three male patients (aged 44, 48, and 60 years) with suspected Brugada syndrome based on Type 2 ECG patterns underwent flecainide challenge testing. One patient received a 400 mg oral dose, while the other two received 200 mg doses. ECG changes were monitored continuously for 24 h. All three patients developed coved-type ST-segment elevation in the right precordial leads (Type 1 Brugada ECG pattern), confirming the diagnosis. The time to onset of diagnostic ECG changes ranged from 15 to 60 min, with peak changes occurring between 90 min and 5 h. No patients experienced syncope, ventricular arrhythmias, or conduction disturbances during or after testing.

**Conclusions:**

This case series suggests that a 200 mg oral flecainide challenge can effectively and safely unmask the diagnostic Type 1 Brugada ECG pattern in selected patients. However, given the small sample size and absence of serum drug concentration data, caution is warranted in interpreting these findings. A lower dose may be a practical alternative to the conventional 400 mg, maintaining diagnostic sensitivity while potentially reducing adverse event risk. Further prospective studies with larger cohorts and longer follow-up are essential to validate the diagnostic performance, safety, and clinical implications of low-dose oral flecainide provocation testing.

## Background

Brugada syndrome (BrS) is an autosomal dominant genetic disorder of the cardiac ion channels, associated with an increased risk of life-threatening ventricular arrhythmias such as ventricular tachycardia (VT) and ventricular fibrillation (VF), which may cause syncope or sudden cardiac death (SCD) in individuals with structurally normal hearts. First described in 1992 by Brugada et al., the syndrome is electrocardiographically defined by incomplete right bundle branch block (RBBB) and ST-segment elevation in the right precordial leads (V1–V3) [1]. However, mounting evidence indicates that BrS is not solely a channelopathy in normal hearts but may also involve underlying myocardial abnormalities [2].

The clinical expression of BrS is often absent at baseline and may require pharmacological challenge to reveal the diagnostic Type 1 ECG pattern. Although previous isolated cases with the characteristic pattern were reported earlier by Andrea Nava and colleagues [3, 4], sodium channel blockers play a crucial role in the diagnostic confirmation of Brugada syndrome by revealing its characteristic Type 1 ECG pattern in patients with indeterminate baseline findings, typically Type 2 patterns. Several pharmacological agents, such as intravenous ajmaline and flecainide, have been widely used for this purpose [5]. Among these, oral flecainide has been evaluated in clinical trials at doses of 400 mg and, more recently, 300 mg, showing high sensitivity in revealing the Brugada ECG pattern [6–8].

Concerns about proarrhythmic effects at higher doses have led to growing interest in lower-dose regimens. However, the efficacy and safety of a 200 mg oral dose have not been systematically studied. To address this gap, we performed provocation testing in three patients, administering 400 mg in one case and 200 mg in two cases, and monitored their clinical and ECG changes over 24 h to assess the diagnostic value of the lower dose (Table 1).

**Table 1:** Diagnostic outcomes of the oral flecainide provocation test in Brugada syndrome

Patient	Flecainide dose (mg)	Time to Type 1 ECG onset (min)	Time to peak ECG changes (h)	Maximum ST elevation (mm)	Adverse events
1	400	30	3	3.5	None
2	200	60	5	6.0	None
3	200	15	5	5.0	None

*ECG* electrocardiogram, *ST* elevation, *ST* segment elevation, *mg* milligrams, *min* minutes, *h* hours, *mm* millimeters

## Case presentation

### Patient 1

A 60-year-old male with a two-year history of hypertension presented with exertional chest pain one day before admission. His highest systolic blood pressure was 180–190 mmHg, but treatment had been irregular. The chest pain was non-radiating, accompanied by heaviness in the left chest, lasting about 10 min and resolving with rest. He also reported palpitations, transient tachycardia, and sweating. He had no history of syncope but occasionally noted rapid heartbeat and palpitations. There was no family history of cardiovascular disease or sudden cardiac death (SCD). BMI was 23.8 kg/m^2^ (height: 175 cm, weight: 73 kg), classifying him as slightly overweight. Laboratory and imaging findings included: normal blood count, echocardiogram, and 24-h ambulatory ECG; normal 128-slice coronary CT angiography (MSCT); mild hypokalemia (3.22 mmol/L); lipid metabolism disorder. A 12-lead ECG showed ~ 2 mm saddleback ST-segment elevation in V1 and V2, consistent with a Type 2 Brugada pattern. Other ECG parameters: PR interval 178 ms, QRS duration 134 ms, corrected QT interval (QTc) 456 ms.

Flecainide challenge test: The patient underwent a provocation test with a single 400 mg oral dose of flecainide following a standardized protocol. The test was conducted in the emergency unit of the Internal Medicine Department, Military Hospital 103, with full resuscitation equipment available. Baseline ECG: no significant ST elevation, PR interval 180 ms, QRS duration 122 ms, QTc 418 ms.

**Fig. 1:**
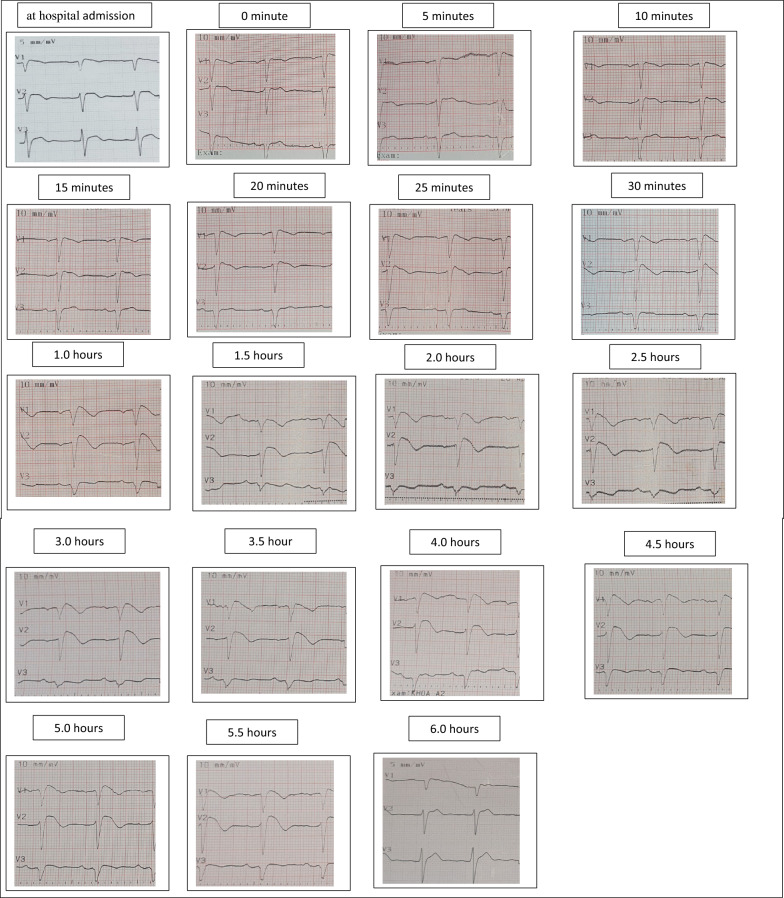
Serial ECG Changes in Patient 1 Following a 400 mg oral flecainide challenge. Serial electrocardiographic (ECG) recordings of Patient 1 during oral flecainide provocation testing (400 mg single dose). V1 and V2 electrodes were placed in the 3rd intercostal space for optimal Brugada pattern detection. Coved-type ST-segment elevation developed 30 min post-administration and reached maximum positivity at 3 h. No biphasic pattern was observed in this case

*ECG monitoring* Serial ECGs were recorded at 5, 10, 15, 20, 25, 30, and 60 min, then every 30 min up to 6 h, with continuous monitoring for 24 h. High right precordial leads (V1 and V2) were recorded in the 3rd intercostal space as recommended for Brugada syndrome evaluation. Coved-type ST-segment elevation appeared in V1–V3 after 30 min. The maximum diagnostic positivity was at 3 h in V2, with an ST elevation of 3 mm. At peak changes, PR interval was 212 ms, QRS duration 148 ms, QTc 490 ms (Fig. 1). No adverse events (e.g., AV block, atrial or ventricular tachycardia) were observed.

*Post-test management* The patient was advised on lifestyle modifications to reduce arrhythmic risk, including: (1) avoiding Brugada triggers (e.g., fever, excessive alcohol, certain medications); (2) regular electrolyte monitoring, especially potassium; (3) routine ECG follow-ups and immediate medical attention for warning symptoms.

### Patient 2

A 44-year-old male with a three-month history of diastolic hypertension (85–95 mmHg) presented with occasional dizziness, palpitations, and mild chest discomfort, particularly during psychological stress. He had not received antihypertensive treatment and denied syncope, dyspnea, or exertional chest pain. There was no family history of SCD or inherited cardiovascular disorders. BMI was 21.1 kg/m^2^ (height: 174 cm, weight: 64 kg). Routine laboratory and cardiac evaluations were normal. A 12-lead ECG showed ~ 2 mm saddleback ST-segment elevation in V1 and V2, consistent with a Type 2 Brugada pattern. Other baseline parameters: PR 178 ms, QRS 134 ms, QTc 456 ms.

*Flecainide challenge test* After specialist consultation, the patient underwent a provocation test with a single 200 mg oral dose of flecainide, following the same protocol as Patient 1. Baseline ECG: no significant ST elevation, PR 140 ms, QRS 90 ms, QTc 389 ms.

**Fig. 2:**
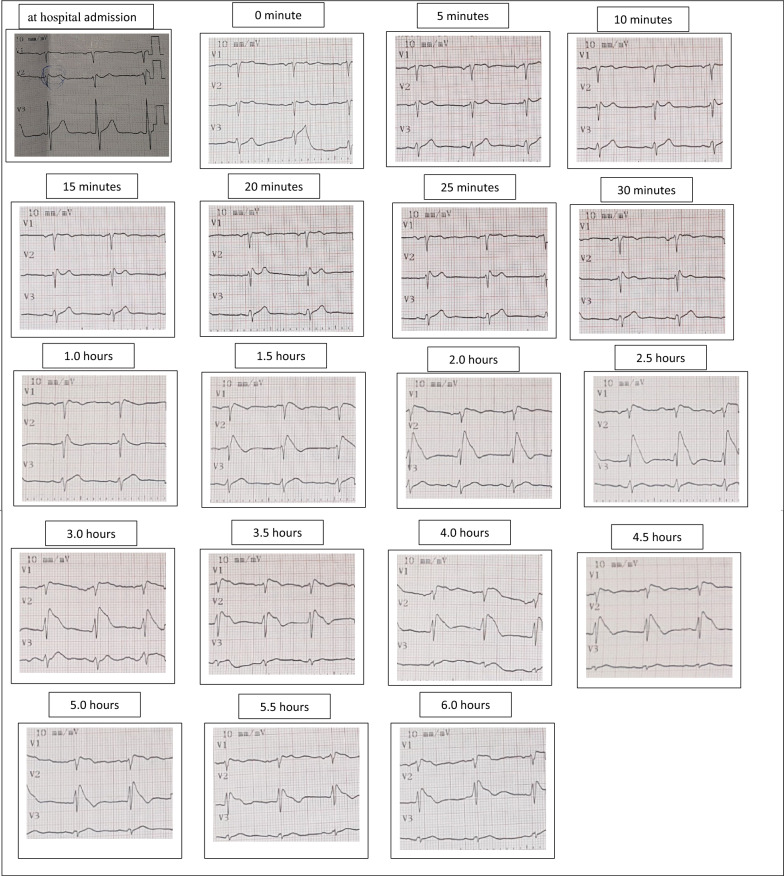
Serial ECG Changes in Patient 2 Following a 200 mg oral flecainide challenge. Serial electrocardiographic (ECG) recordings of Patient 2 during oral flecainide provocation testing (200 mg single dose). V1 and V2 electrodes were placed in the 3rd intercostal space for optimal Brugada pattern detection. Coved-type ST-segment elevation developed at 60 min post-administration, with an initial rise at 2 h followed by a second, higher peak at 5 h, reaching an ST elevation of 6 mm in V2. No ventricular arrhythmias or conduction abnormalities were observed during the test

*ECG monitoring* Serial ECGs were obtained as above, with high right precordial leads recorded in the 3rd intercostal space. Coved-type ST elevation appeared in V1–V2 at 60 min, showing a biphasic pattern—an initial peak at 2 h, partial decline, and a second peak at 5 h with maximum STE of 6 mm in V2. Peak ECG: PR 156 ms, QRS 94 ms, QTc 405 ms (Fig. 2). This biphasic response highlights the importance of extended ECG monitoring after oral flecainide. No adverse events occurred.

*Post-test management* The patient was advised to avoid Brugada triggers, undergo regular cardiac monitoring, and maintain a healthy lifestyle.

### Patient 3

A 48-year-old male presented with recurrent chest pain and was admitted for further evaluation. Since April 2022, he had four episodes of left-sided chest pain, each lasting 5–10 min and occurring after excessive alcohol consumption. The pain was non-radiating, self-limited, and not associated with syncope. He had no known cardiovascular disease and no family history of SCD or inherited arrhythmias. BMI: 20.8 kg/m^2^ (height: 170 cm, weight: 60 kg). Cardiac workup was normal. Baseline ECG showed a Type 2 Brugada pattern with RBBB and ~ 1 mm ST-segment elevation in V1 and V2 (PR 170 ms, QRS 94 ms, QTc 409 ms).

Flecainide challenge test: The patient received a single 200 mg oral dose, using the same monitoring protocol as above. Baseline: no significant ST elevation.

**Fig. 3:**
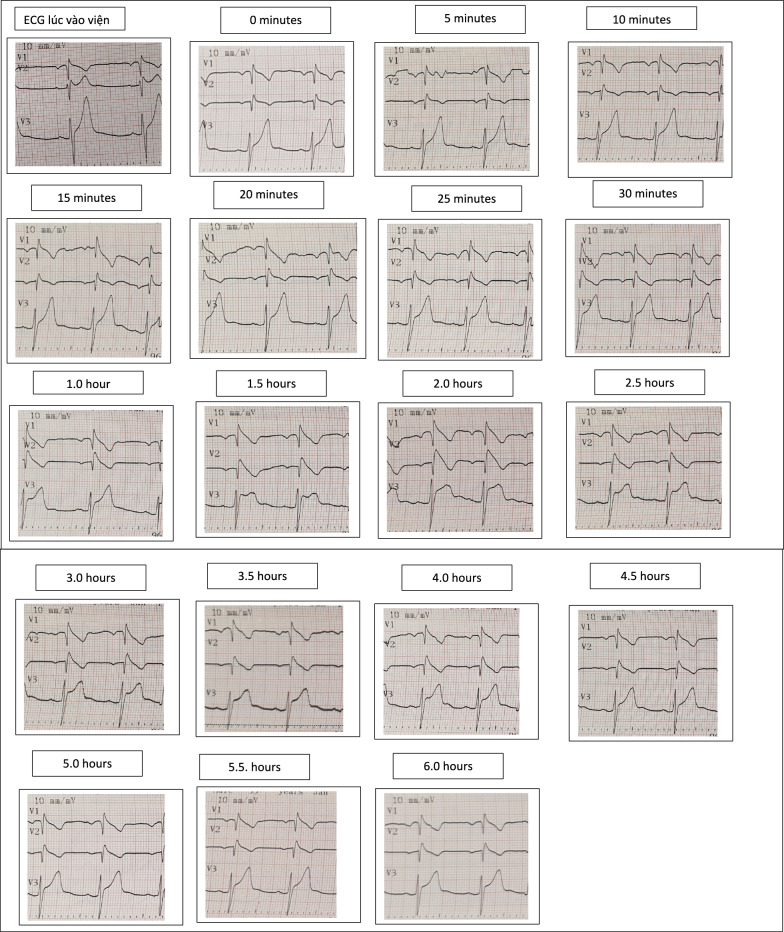
Serial ECG Changes in Patient 3 Following a 200 mg oral flecainide challenge. Serial electrocardiographic (ECG) recordings of Patient 3 during oral flecainide provocation testing (200 mg single dose). V1 and V2 electrodes were placed in the 3rd intercostal space for optimal Brugada pattern detection. Coved-type ST-segment elevation developed at 15 min post-administration and reached maximum positivity at 2 h, with an ST elevation of 5 mm in V3. No ventricular arrhythmias or conduction abnormalities were observed throughout the test

ECG monitoring: Serial ECGs were recorded with high right precordial leads in the 3rd intercostal space. Coved-type ST elevation appeared in V2 at 15 min, peaking at 5 h in V3 with STE 5 mm. Peak ECG: PR 208 ms, QRS 112 ms, QTc 442 ms (Fig. 3). No arrhythmias or conduction blocks occurred.

*Post-test management* Advice was similar to previous patients.

## Discussion

### Electrocardiographic findings and flecainide provocation testing

In patients with suspected but inconclusive Brugada syndrome (BrS), particularly those showing a Type 2 ECG pattern, the first recommended step is to record high precordial leads (V1 and V2 in the 2nd or 3rd intercostal space). This maneuver, described by Márquez et al. [9], and confirmed in pediatric populations by Manzano-Cabada et al. [10], can unmask a diagnostic Type 1 ECG without pharmacological challenge. In our protocol, V1 and V2 were placed in the 3rd intercostal space, in accordance with European guidelines, to maximize diagnostic yield. Practical considerations such as patient compliance and body habitus may also influence electrode placement.

When high precordial leads fail to reveal the pattern, pharmacological challenge becomes the next step. Standard agents include ajmaline, procainamide, flecainide, and pilsicainide. Flecainide, a Class 1C antiarrhythmic, blocks fast sodium (Na⁺) channels during phase 0 of the cardiac action potential, slowing conduction velocity, widening QRS duration, and altering repolarization. It also inhibits late Na⁺ currents, delayed rectifier potassium (K⁺) currents, and ryanodine receptor activity, enhancing epicardial-to-endocardial voltage gradients and producing ST-segment elevation in V1–V3 [11].

### ECG response and dose considerations

Oral flecainide is highly bioavailable (80–90%), with peak plasma concentrations within 1–4 h and dose-proportional pharmacokinetics [12]. In this study, due to the lack of intravenous options, we administered 400 mg in one patient and 200 mg in two patients, following current recommendations. Previous reports with 300–400 mg doses showed no serious arrhythmias [7, 13], and we similarly observed no conduction disturbances or ventricular arrhythmias after excluding structural heart disease.

The onset of ST-segment elevation ranged from 15 to 60 min, peaking at 3 h (Patient 1, 400 mg), 5 h (Patient 2, 200 mg, biphasic pattern), and 5 h (Patient 3, 200 mg). Interestingly, one patient receiving 200 mg developed positivity earlier than the 400 mg case, suggesting individual differences in drug metabolism and ion channel sensitivity. This observation supports the potential of a lower dose to achieve diagnostic yield while possibly reducing proarrhythmic risk.

The variation in time to peak ECG changes (90 min to 5 h) likely reflects differences in absorption, metabolism, autonomic tone, and ion channel sensitivity [12]. This underlines the importance of serial ECG monitoring over several hours to avoid false-negative results, particularly with oral formulations.

### Comparison with previous studies and safety

Sergio Dubner et al. [13] reported 50% positivity for Type 1 Brugada pattern using 400 mg oral flecainide, with modest PR, QRS, and QTc prolongation and no arrhythmias. Our results with 200 mg are comparable, suggesting that a lower dose can preserve diagnostic sensitivity while reducing potential proarrhythmic risk [14], Safety precautions remain essential—patients should be monitored continuously with resuscitation equipment on hand, especially in those with baseline conduction abnormalities or unexplained syncope [12].

### Risk stratification in Brugada syndrome

Risk assessment remains challenging, especially for asymptomatic individuals with drug-induced Type 1 patterns. High-risk features include prior arrhythmic syncope, spontaneous Type 1 ECG, early repolarization in peripheral leads, and Type 1 pattern in peripheral leads [15]. Models such as the PAT score [16, 17] attempt to predict arrhythmic risk but have limitations in asymptomatic patients. Meta-analyses suggest low conversion rates to spontaneous Type 1 in drug-induced cases during follow-up [18], supporting conservative management in selected patients..

### Prevention strategies for ventricular arrhythmias and SCD

Preventing arrhythmic events in BrS focuses on avoiding known triggers and maintaining protective measures: (1) Fever control—Fever can accentuate Brugada ECG changes and increase arrhythmic risk; patients should use antipyretics promptly. (2) Avoiding proarrhythmic drugs—Sodium channel blockers, beta-blockers, certain antidepressants, lithium, and some antipsychotics should be avoided (full list at www.brugadadrugs.org). (3) Limiting alcohol intake—Alcohol has been associated with increased arrhythmic risk. (4) Avoiding stimulants—Cocaine, certain energy drinks, and large meals may provoke arrhythmias. (5) Nutritional considerations—While dietary measures alone are insufficient for arrhythmia prevention, some nutrients may have cardioprotective effects (D'Imperio S et al.) [19]. An emerging concern is the psychological burden in young, asymptomatic patients after a BrS diagnosis, particularly when no active therapy is offered. The term “Brugadaphobia” describes persistent fear, anxiety, and health-related rumination following a positive test (Viskin et al.) [20]. Addressing this requires pre-test counseling, shared decision-making, and long-term psychological support.

### Screening and genetic testing in family members

Supporters point to the value of early detection and cascade screening, but recent evidence indicates that drug-induced Type 1 ECG without spontaneous pattern or symptoms carries a low arrhythmic risk (Gaita et al.) [21], (Russo et al.) [22]. As Viskin et al. (2024) emphasize, testing should be considered selectively, taking into account clinical context, family history, and psychological readiness. Given the genetic nature of BrS, all first-degree relatives of diagnosed patients should undergo: Clinical examination; Baseline and, if indicated, provocation ECG testing; Genetic testing (if available) (Y. G. Antzelevitch et al.) [6]. Early identification allows closer monitoring, patient education, and potentially life-saving interventions, especially in asymptomatic carriers with latent or inducible Type 1 ECG patterns.

### Limitations

This study has several limitations. First, the very small sample size (three cases) limits generalizability and prevents statistical comparison between doses. Second, serum flecainide concentrations were not measured, preventing direct correlation between drug exposure and ECG response. Third, potential selection bias may exist, as patients undergoing provocation testing were carefully screened and may not represent the broader Brugada population. Fourth, inter-individual variability in gastrointestinal absorption, hepatic metabolism, and cardiac electrophysiology may have influenced the observed differences in response timing and ST-segment changes. Finally, ECG monitoring was limited to 24 h, which may not capture delayed arrhythmic events or late spontaneous conversion to a diagnostic Type 1 pattern. These factors highlight the need for larger, prospective studies directly comparing the 200 mg dose with higher doses to define the optimal balance between diagnostic yield and safety.

## Conclusion

This case series suggests that a 200 mg oral flecainide challenge can unmask the diagnostic Type 1 Brugada ECG pattern in selected patients and may be a safer alternative to higher doses. While this lower dose could reduce proarrhythmic risk, the findings are preliminary. Larger comparative studies are required to confirm efficacy, safety, and optimal dosing before routine clinical use.

## Data Availability

No datasets were generated or analysed during the current study.

## Abbreviations

Ajm: Ajmaline
AV block: Atrioventricular block
bpm: Beats per minute
BrS: Brugada syndrome
ECG: Electrocardiogram
Flec: Flecainide
ICD: Implantable cardioverter-defibrillator
IV: Intravenous
JACC: Journal of the American College of Cardiology
K⁺: Potassium ion
Ms: Milliseconds
Na⁺: Sodium ion
QTc: Corrected QT interval
RBBB: Right bundle branch block
SCD: Sudden cardiac death
SCN5A: Sodium channel, voltage-gated, type V, alpha subunit
V1–V3: Precordial leads V1 to V3
VF: Ventricular fibrillation
VT: Ventricular tachycardia
